# Cognitive Impairment in a 64-Year-Old Male: Dilemmas With Differential Diagnosis for Patients With Dementia

**DOI:** 10.7759/cureus.55024

**Published:** 2024-02-27

**Authors:** Eduardo D Espiridion, Noorvir Kaur

**Affiliations:** 1 Psychiatry, Drexel University College of Medicine, Philadelphia, USA; 2 Psychiatry, Reading Hospital, West Reading, USA; 3 Psychiatry, Drexel University College of Medicine, West Reading, USA

**Keywords:** memory, mental status change, confusion, cognitive impairment, dementia

## Abstract

Dementia is characterized by cognitive impairment and difficulties in executive functioning. It is an umbrella term for different subtypes that should be differentiated using a meticulous review of the patient’s history, physical exam, and work-up. Posing difficulties in diagnosis, findings at times may be inconclusive. We report a case of a depressed patient on hemodialysis for end-stage renal disease (ESRD) who presents with an acute agitated episode following a visual hallucination that he has been experiencing intermittently for six months, along with a three- to four-year history of cognitive impairments. Our differential diagnosis includes vascular dementia, dementia with Lewy bodies, pseudodementia, dialysis dementia, and early-onset Alzheimer’s. In this case, the findings of a normal mini-mental status exam (MMSE) and mental status exam (MSE) do not correlate with a working diagnosis. Due to persistent dilemmas in diagnosing early neurocognitive impairment, continued monitoring and re-assessment are necessitated for efficient management of psychiatric patients with cognitive decline.

## Introduction

Dementia is used to describe progressive and unrelenting deterioration of mental capacity [1], defined as a state of impairment in memory with at least one other domain of cognitive function, such as language, orientation, reasoning, attention, or executive functioning [2]. Its prevalence is 15% in individuals older than 68 years [3]. Risk factors for all-cause dementia include increasing age and female sex, as well as protective factors such as higher education and cognitive or physical activity. Clinical presentation varies greatly with cognitive deficits of memory loss, communication, and language impairments, and in severe cases, aphasia, agnosia, and apraxia [4]. Dementia is an umbrella term with subtypes of Alzheimer’s dementia (AD), vascular dementia, Lewy body dementia, and frontotemporal dementia. Despite advances in clinical practice with evidence-based assessment scales, difficulties in diagnosing the cause of dementia or its subtypes remain apparent. Older persons tend to have more etiologies compared to younger individuals, which attenuates the accuracy of the diagnosis [5].

Other less commonly seen subtypes should be considered as well, such as pseudodementia and dialysis dementia. Pseudodementia is a term coined to signify cognitive and functional impairment that imitates neurodegenerative disorders secondary to uncontrolled major depressive disorder [6]. Patients with pseudodementia typically have equal loss of recent and remote events, are associated with patchy or specific memory loss, and give frequent “don’t know” answers but have intact attention and concentration [7]. One population-based study that recruited patients from primary care practices found depressive pseudodementia in 0.6% of people aged 65 years or older [8].

Dialysis dementia is slow and progressive in onset and is caused by aluminum contamination. It occurs in dialysis units that do not use reverse osmosis or deionization techniques and/or with aluminum-containing medications in oral phosphate binders [9]. Initial symptoms include dysarthria, apraxia, and slurring of speech with stuttering and hesitancy. Later in the course of the disease, symptoms progress to personality changes, psychosis, myoclonus, seizures, and eventually dementia and death within six months after the onset of symptoms [9]. 

Despite seemingly disparate classical descriptions, there is significant overlap between the presentations in clinical practice [10]. Several previous studies assessed clinical diagnostic quality for dementia subtypes, including one that measured the mismatch rate between clinical diagnosis and post-mortem neuropathological results of seven dementia subtypes, finding the clinical misdiagnosis rate high among all subtypes [10]. Inaccuracy or delayed recognition of dementia subtypes can prove to be detrimental and negatively impact patient outcomes.

## Case presentation

A 64-year-old male with a past psychiatric history of depression and anxiety was brought to the emergency department (ED) of a community hospital by emergency medical services (EMS) due to altered mental status for one day. Prior to arrival, the patient had an episode of combativeness and aggression with nursing staff at the assisted care facility, in which he felt he was moved to a different room and became angry. Upon arrival, the patient was calm and cooperative. He endorsed a history of visual hallucinations of seeing windows and doors in a room and of him playing ball or pool for approximately six months without auditory hallucinations. Collateral information revealed an approximately four-year history of cognitive impairment and worsening depression, with increased difficulty in completing his activities of daily living (ADL). He was previously hospitalized two months prior for a change in mental status following hemodialysis treatment. The work-up at that time revealed no abnormalities and was attributed to the patient's medications of methocarbamol and oxycodone, for which the oxycodone dosage was decreased by half to 2.5mg.

Upon examination, the patient presented with hypertensive urgency and a blood pressure of 219/105, for which labetalol 10 mg was administered, with the rest of his vital signs within normal limits. He responded to questions appropriately, with no notable dysarthria. He followed simple commands with no apraxia. His neurological exam was unremarkable. The patient’s mood was described as “okay,” and he demonstrated a constricted affect. He was not aphasic, and his speech was coherent.

Given the acuity of presentation, electrolyte imbalances such as hyponatremia, hypercalcemia, hypoglycemia, infectious causes of delirium, and reversible causes of dementia such as syphilis, HIV, and hypothyroidism were to be ruled out. The key laboratory findings are presented in Table 1. We also looked into vitamin deficiencies to rule out reversible causes of dementia, and his vitamin B12 and folic acid were within normal limits. Medication-induced delirium was also considered by the medical team, and the patient’s oxycodone and gabapentin were held. However, his home medications of escitalopram 20 mg and trazodone 25 mg nightly were continued.

**Table 1:** Relevant laboratory findings GFR: glomerular filtration rate

Laboratory test	Results	Reference values
Serum sodium	137 mmol/L	136 - 145 mmol/L
Serum potassium	4.3 mmol/L	3.5 - 5.1 mmol/L
Serum calcium	9.7 mg/dL	8.7 - 10.4 mg/dL
Ionized calcium	1.11 mmol/L	1.15 - 1.33 mmol/L
Serum creatinine	6.08 mg/dL	0.73 - 1.18 mg/dL
Blood urea nitrogen (BUN)	29 mg/dL	9 - 23 mg/dL
Phosphorus	4.8 mg/dL	2.4 - 5.1 mg/dL
Estimated GFR Chronic Kidney Disease Epidemiology Collaboration eGFR (CKD-EPI)	9.6 mL/min/1.73m*2	>60 mL/min/1.73m*2
Thyroid function test	1.942 ulU/mL	0.550 - 4.780 ulU/mL
Glucose	143 mg/dL	74 - 99 mg/dL
Hemoglobin	13.2 g/dL	14.0 - 17.5 g/dL
Hematocrit	39.40%	39.0% - 53.0%
White blood cell count	7.0 x 109/L	4.8 - 10.8 x 109/L
Folic acid	> 24.0 ng/mL	>5.4 ng/mL
Vitamin B12	454 pg/mL	211 - 911 pg/mL
Treponemal antibody	Non-reactive	Non-reactive
HIV antigen/antibody, 4th generation	Non-reactive	Non-reactive

**Figure 1:**
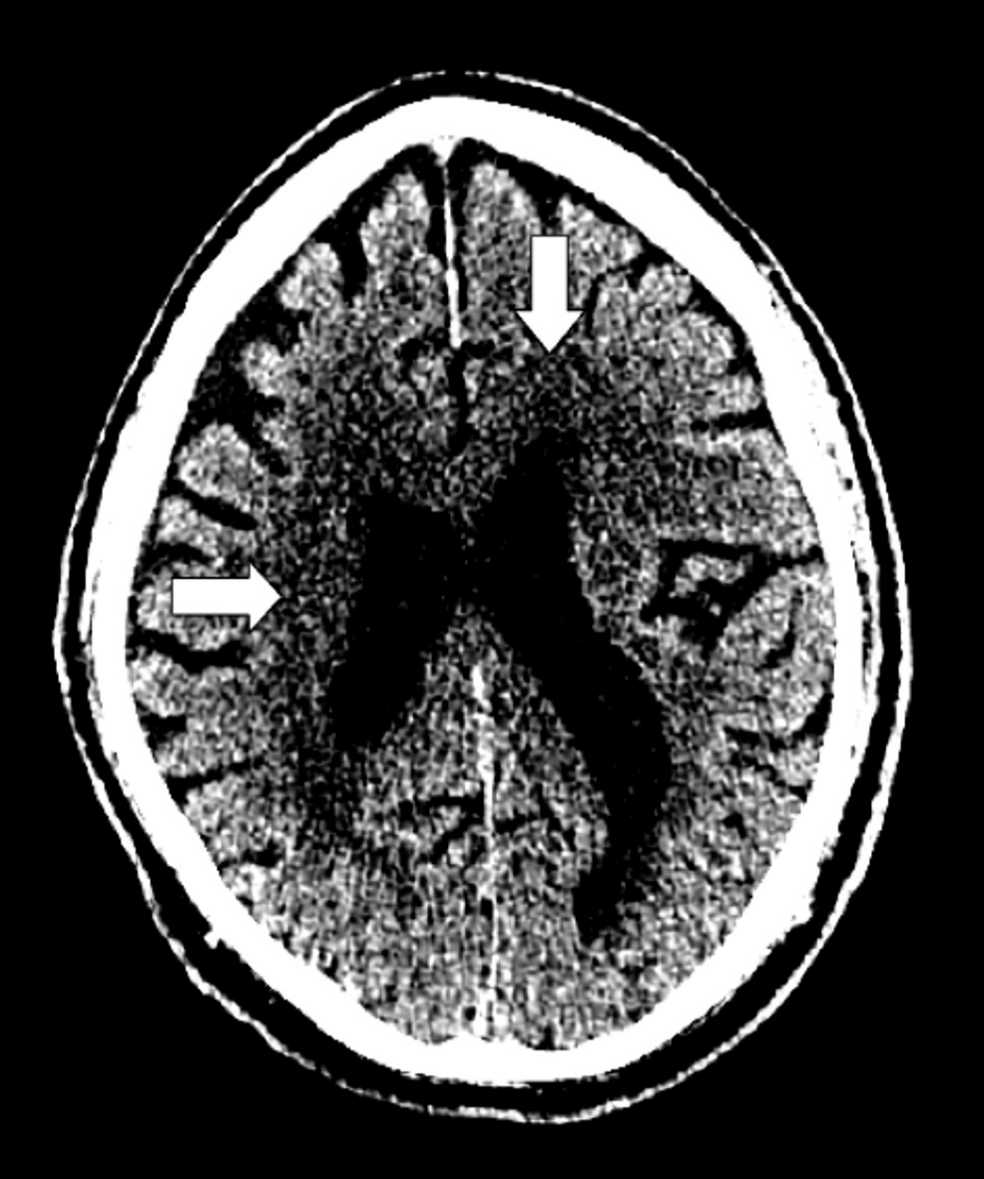
Thin standard view of the CT brain without contrast demonstrates diffuse periventricular and subcortical white matter hypodensities as indicated by the white arrows

**Figure 2:**
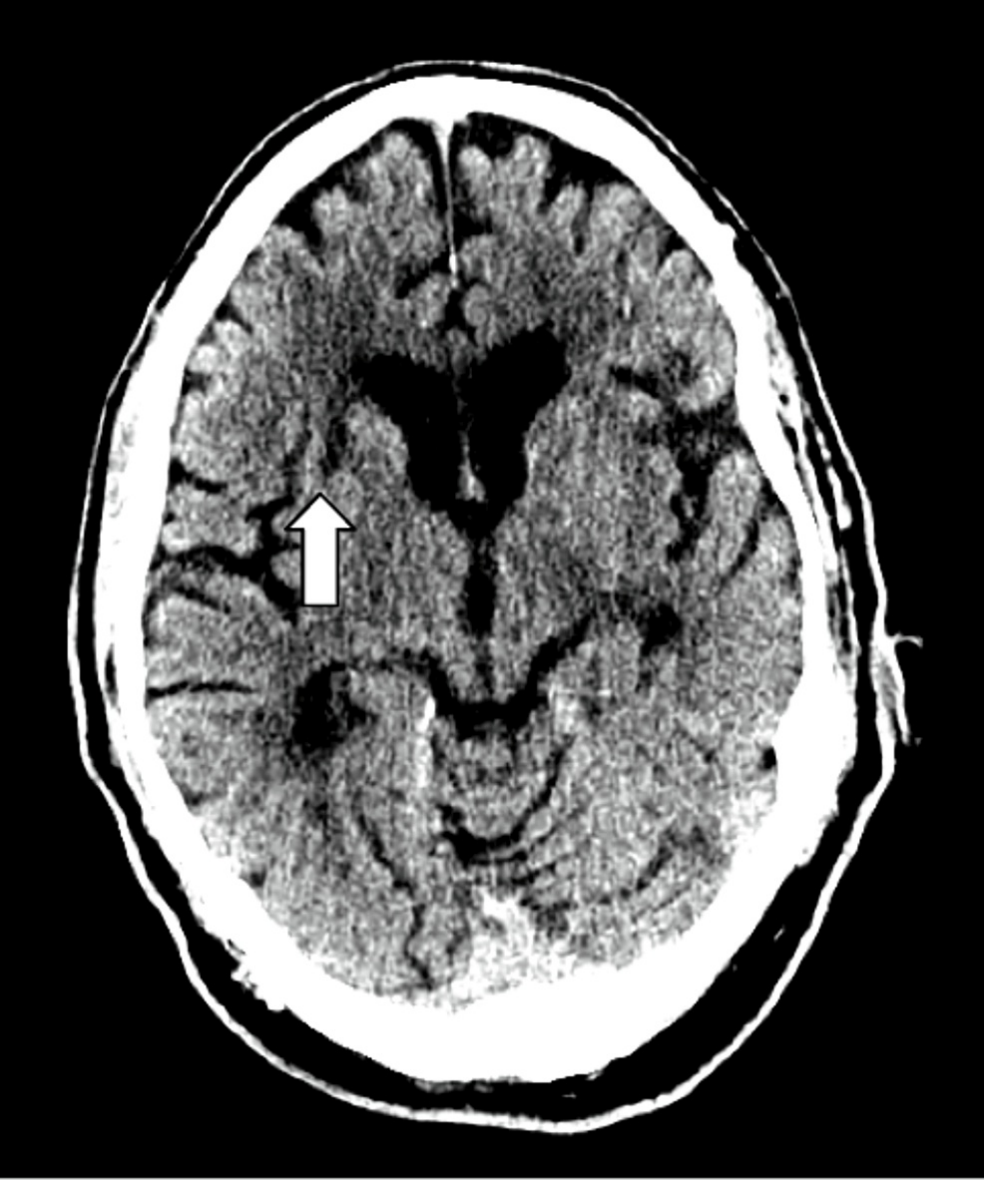
Standard view of CT brain without contrast demonstrating right lacunar infarct within basal ganglia as indicated by the white arrow

Imaging studies of the CT brain showed changes in scattered periventricular and subcortical white matter hypodensities, most consistent with chronic small vessel ischemic disease, as evidenced by Figures 1, 2. Right frontal encephalomalacia was also notably unchanged from previous studies, consistent with the patient's history of ischemic multifocal multiple vascular territory stroke, and no evidence of acute hemorrhage was reported. Given impairment in cognitive functioning coupled with impairments in executive functions, potential etiologies for the patient’s dementia included vascular impairment or dementia, dementia of Lewy bodies, pseudodementia, dialysis dementia, and early-onset Alzheimer’s. 

After his electrolyte abnormalities were corrected, the patient continued to have cognitive impairment. It was noted that his attention span had improved, but he continued to have short-term memories, including his inability to recall important dates like the birthday of his daughter. Psychiatry evaluated the patient during their hospital stay and gave them quetiapine as needed for agitated episodes. An MMSE was administered, and the patient scored 28 out of 30 points; he could not recall the day of the year or draw the intersecting pentagons. The patient did ultimately report episodes of hypnopompic hallucinations, although without daytime somnolence, sleep attacks, or cataplexy. An outpatient polysomnography test, followed by a sleep latency test, was to be arranged to evaluate for narcolepsy and rapid eye movement (REM) sleep disorders. During his hospital stay, he received hemodialysis and was noted to have intradialytic hypotension, which resolved after a normal saline bolus, but he did not report any episodes of subsequent altered mental status. An electroencephalogram (EEG) was not performed for this patient. This work-up may rule out ongoing delirium in the differential diagnoses of cognitive impairment, especially for this patient, who has presented with delirium in the past. A follow-up mini-mental status exam (MMSE) was to be performed to determine the persistence of his cognitive impairment, given that he has cognitive deficits. However, this was not done as the patient was discharged from the hospital.

## Discussion

The diagnosis of dementia subtypes can be particularly challenging with early cognitive changes due to similar presenting symptoms and overlapping neuropsychiatric profiles. In this case, specific criteria were to be considered to delineate between them. The most likely diagnosis would be vascular impairment or dementia, as it presents with stepwise cognitive decline in at least one cognitive domain [1], alongside supportive imaging evidence of subcortical and microvascular changes. The patient’s risk factors of diabetes and hypertension, given that the patient presented with a hypertensive crisis, further support this, as these can cause arteriolosclerosis of long, penetrating vessels, causing chronic ischemia of periventricular and deep white matter and microvascular disease, respectively. Cognitive decline was also found to progress faster in patients with end-stage renal disease (ESRD) than in those without, such that by the time patients are established on dialysis, >30% have severe cognitive impairment consistent with dementia [3]. The patient’s CT findings further support findings of chronic small vessel disease consistent with a vascular dementia etiology. Further neuropsychiatric testing should focus on elucidating subtle executive function deficits to suggest a subcortical ischemic vasculopathy.

Synucleinopathies like dementia with Lewy bodies (DLB) are possible, given the visual hallucinations the patient was experiencing and fluctuating changes in cognition. However, he did not overtly display Parkinsonian features or describe a prior history of REM sleep behavior disorders. Furthermore, visual hallucinations in DLB tend to be well-formed, feature people or animals, and are usually of different sensory modalities [11]. Rating scales, including collateral information regarding symptomatology, would be essential to diagnosis and management [12]. Further diagnostic markers could be sought out, such as DLB biomarkers or ligands or the dopamine transporter, a volumetric analysis of an MRI scan to demonstrate putamen/dorsal mesopontine gray matter atrophy, and an EEG for prominent posterior slow wave activity [13,14], alongside an outpatient polysomnography sleep study.

Given the patient's worsening depression, pseudodementia could also be possible, which is prevalent in the elderly population. However, he has been on maintenance therapy with escitalopram and reported no sleep or appetite disturbances. Depression may also present with several memory-function deficits, which are more common with tasks of recall or remembering [7], and the patient performed well in recall tasks on the MMSE, although a single screening tool should be followed with subsequent screenings. To further evaluate this, the Cornell Scale for Depression in Dementia (CSDD) evaluates mood-related signs and behavioral disturbances in a 19-item questionnaire, which could be pursued in this case [6]. For further work beyond the MMSE and mental status exam (MSE), routine psychometric testing at outpatient follow-up between nine and twelve months may be pursued for a well-informed diagnosis. When the history suggests cognitive impairment but the examination is relatively normal, possible explanations include mild dementia, high intelligence or education, and depression.

With the patient receiving hemodialysis for approximately three years, dialysis dementia should be considered, especially with a previous altered mental status episode following a previous hemodialysis treatment. With the onset of improved water treatments, dialysis dementia has become rarer since the 1980s. This is also less likely, as he did not experience speech changes, involuntary jerking, or seizure-like activity. In cases of suspected dialysis dementia, serum aluminum levels should be obtained, as a level > 80 μg/L has been associated with dialysis encephalopathy [9]. Periodic screening (every 12 months) is important for recognizing cognitive impairment in ESRD that develops over time. Early-onset Alzheimer’s would be characterized by progressive, episodic memory impairments with later deficiency of language and visuospatial abilities [5], but less likely within this case given no prior imaging findings suggestive of diffuse cortical or hippocampal atrophy [13]. Alzheimer’s is particularly difficult to diagnose in the early stages as its testing has low sensitivity and specificity. While sensitivity improved at more advanced dementia stages, its specificity dropped [10]. 

Although the MMSE and neurologic exam may have been benign, the leading diagnosis remains vascular dementia, which can be deduced from the imaging studies and history within the framework of early cognitive decline. Vascular dementia is a neurocognitive, nondegenerative disease that, in a patient with early cognitive decline, may lead to further functional status decline paired with focal neurological deficits. Previous reports may underestimate or not delineate the importance of continued monitoring for a temporal or chronological understanding of a patient’s cognitive decline. Therefore, methodical follow-up of labs such as biomarkers, rating scales, and outpatient studies should be corroborated with previous records of cognitive impairment to fully understand a patient’s dementia subtype.

## Conclusions

The complex interplay of diagnosing early neurocognitive decline with an incongruent history and exam findings can be difficult. It is salient to understand the similarities but, more importantly, the differences in common and uncommon, irreversible causes of dementia through effective history-taking, collateral information, and thorough clinical investigations to increase diagnosis efficiency and augment appropriate interventions. This case demonstrated a meticulous review of differential diagnosis but also emphasizes the importance of a thorough work-up and follow-up protocol to avoid underdiagnosis or misdiagnosis of a dementia subtype.
